# Diagnostic and Prognostic Value of IL-6 and sTREM-1 in SIRS and Sepsis in Children

**DOI:** 10.1155/2020/8201585

**Published:** 2020-06-22

**Authors:** Beata Smok, Krzysztof Domagalski, Małgorzata Pawłowska

**Affiliations:** ^1^Department of Infectious Diseases and Hepatology, Faculty of Medicine, Collegium Medicum Nicolaus Copernicus University, Poland; ^2^Department of Immunology, Faculty of Biological and Veterinary Sciences, Nicolaus Copernicus University, Poland

## Abstract

**Purpose:**

The aim of this study was to evaluate the diagnostic and prognostic value of IL-6 and sTREM-1 in the course of SIRS and sepsis in children with reference to routinely used CRP and PCT.

**Methods:**

A prospective study included 180 patients at the ages from 2 months to 18 years hospitalized due to fever from November 2015 to January 2017. Forty-nine children without fever hospitalized due to noninfectious causes formed the control group. IL-6 and sTREM-1 serum concentrations were assessed with the enzyme-linked immunosorbent assay method.

**Results:**

The mean serum concentrations of all the analyzed biomarkers were statistically significantly higher in the study group compared to the control group. Mean IL-6, sTREM-1, and PCT serum concentrations were statistically significantly higher in the group of patients with SIRS/sepsis compared to the group of feverish patients without diagnosed SIRS (N-SIRS). Based on the ROC curve analysis, it was shown that of all the biomarkers tested, only two—IL-6 and procalcitonin—had potential usefulness in the diagnosis of SIRS/sepsis in children with fever.

**Conclusion:**

Elevated levels of IL-6 and PCT are important risk factors for the development of SIRS/sepsis in children with fever. It seems that elevated IL-6 baseline serum level may predict a more severe course of febrile illness in children, because based on the ROC curve analysis, it was found that IL-6 is a statistically significant prognostic marker of prolonged fever ≥ 3 days and prolonged hospitalization > 10 days. The assessment of the usefulness of sTREM-1 in the diagnosis of SIRS/sepsis in feverish children requires further research.

## 1. Introduction

Systemic inflammatory response syndrome (SIRS) and sepsis are common causes of morbidity among children. Early diagnosis and immediate initiation of proper treatment have a major impact on improving the prognosis in these patients. The American College of Chest Physicians (ACCP) and Society of Critical Care Medicine (SCCM) established definitions for sepsis, severe sepsis, and septic shock, while introducing the definition of SIRS in 1992 [1]. In clinical practice, especially the SIRS criteria proved to be not specific enough [2]. The current guidelines were announced in 2016 as the Third International Consensus Definitions for Sepsis and Septic Shock. Limitations of previous definitions included an excessive focus on inflammation, the misleading model that sepsis follows a continuum through severe sepsis to shock, and inadequate specificity and sensitivity of the systemic inflammatory response syndrome criteria [3].

Markers used routinely in diagnostics such as C-reactive protein (CRP) and procalcitonin (PCT) are also not sufficiently sensitive and specific [4–8]. Therefore, new markers of acute inflammation, which would be a useful diagnostic and prognostic tool in children with clinical suspicion of SIRS and sepsis, are being studied. These include interleukin-6 (IL-6) and sTREM-1 (soluble triggering receptor expressed on myeloid cells-1).

Interleukin-6 is one of the major proinflammatory cytokines. It exerts a significant influence on the innate immune cells, increasing the sensitivity of monocytes and neutrophils as well as cytotoxic activity of NK cells. At the same time, it affects the humoral and cellular adaptive immunity response. IL-6-secreting cells include both cells of the immune system—monocytes, lymphocytes T and B, neutrophils, and NK cells—and a number of other cells, such as keratinocytes, fibroblasts, endothelial cells, or muscle cells [9]. The physiological serum concentration of IL-6 is 1-5 pg/ml and increases in the course of both infectious and noninfectious conditions, such as trauma, surgery, myocardial infarction, and stroke as well as autoimmune and neoplastic diseases [10–12]. An increase in the concentration of IL-6 in the serum is observed 1-3 hours after the triggering factor [13].

TREM-1 (triggering receptor expressed on myeloid cells-1) was identified by Swiss researchers in 2000 as a novel transmembrane protein belonging to the immunoglobulin superfamily selectively expressed on blood neutrophils, monocytes, and macrophages and is upregulated by bacterial and fungal stimuli. It seems to play a relevant role in the modulation of innate immunity, amplifying or attenuating Toll-Like Receptor- (TLR-) induced signals [14]. TREM-1 interacts with the adaptor protein DAP12 for signaling and function. Engagement of TREM-1 results in the production of chemokines such as IL-8 and proinflammatory cytokines TNF-*α* and IL-1*β* [14–16].

A soluble form of TREM-1 (sTREM-1) is released from the activated phagocytes and can be found in body fluids, such as plasma, pleural fluid, bronchoalveolar lavage fluid, urine, and cerebrospinal fluid. Thus, sTREM-1 may act as a potential biomarker of bacterial infection [14, 17]. Many researchers believe that serum sTREM-1 is not observed in the course of noninfectious inflammatory diseases, suggesting high specificity of sTREM-1 as a biomarker of infection [13, 18–20]. However, recent studies indicate the participation of TREM-1 in the pathogenesis of some acute and chronic noninfectious inflammatory diseases, such as atherosclerosis, inflammatory bowel disease, tissue damage associated with ischemia and reperfusion, or cancer [21].

The aim of this study was to evaluate the diagnostic and prognostic value of IL-6 and sTREM-1 in the course of acute inflammation among children, especially taking into consideration SIRS and sepsis with reference to routinely used CRP and PCT.

## 2. Material and Methods

A total of 180 patients at the ages from 2 months to 18 years who were hospitalized due to fever in the Pediatric Infectious Diseases and Hepatology Ward from November 2015 to January 2017 were subjected to the study. Forty-nine children without fever who were hospitalized due to noninfectious causes formed the control group. Written informed consent was obtained from the parents.

The exclusion criteria were age under one month and over 18 years, acute and chronic liver diseases, chronic inflammatory diseases, malignancy, and congenital and acquired immunodeficiency.

In the diagnosis of SIRS and sepsis, the criteria of the American College of Chest Physicians (ACCP) and the Society of Critical Care Medicine (SCCM) in 1992 [1], modified for children at the International Pediatric Sepsis Conference in 2005, were used [22]. SIRS was diagnosed when, in addition to fever (body temperature above 38.5 degrees Celsius), at least one of the following three criteria was met: (1) tachycardia—mean heart rate per minute over 2 SD (statistical deviation) above age norm, (2) tachypnoea—mean number of breaths per minute over 2 SD above age norm, and (3) leukocyte counts above or below normal for age or above 10% of immature neutrophils. Sepsis was diagnosed in children with systemic inflammatory response syndrome related to infection.

Patients in the study group were divided depending on the severity of the disease into three subgroups: patients who met the SIRS criteria (without sepsis)—45/180 (25%), children who were diagnosed with sepsis—53/180 (29%), and other feverish patients who did not fulfill the diagnostic criteria of systemic inflammatory response syndrome (N-SIRS)—82/180 (46%). None of the children were diagnosed with severe sepsis or septic shock.

The following tests were carried out in all patients at the time of admission: IL-6, sTREM-1, PCT, and CRP serum levels. IL-6 and sTREM-1 serum concentrations were assessed with the enzyme-linked immunosorbent assay method (Quantikine ELISA Human IL-6 Immunoassay, R&D Systems; Human sTREM-1 ELISA Kit, Elabscience). The minimum detection threshold was 0.70 pg/ml for IL-6 and 18.75 pg/ml for sTREM-1.

Statistical analyses were performed using the SPSS program (version 20) and Microsoft Excel. Quantitative data with normal distributions are presented as mean ± standard deviation (SD) and those with nonnormally distributed variables as median with interquartile range (IQR). Qualitative variables were presented as numbers (*n*) and percent (%). Differences in the distribution of qualitative variables were evaluated using Pearson's *χ*^2^ test. Student's *t*-test or the Mann-Whitney *U* test were used for comparison of quantitative variables between two independent groups. For comparing more independent groups, one-way analysis of variance (ANOVA) or the Kruskal–Wallis test as a nonparametric equivalent was used. In order to determine the diagnostic and prognostic value of the studied inflammatory markers, the Receiver Operating Characteristic (ROC) method was used, analyzing the area under the curve (AUC) as well as the sensitivity, specificity, positive predictive value (PPV), and negative predictive value (NPV). A *p* value < 0.05 was considered statistically significant.

## 3. Results

Demographic and clinical characteristics in the study and control groups are presented in Table 1. Among all patients included to the study, there were 129/229 (56.3%) boys and 100/229 (43.7%) girls. The mean age was 6.4 for the study group and 7.1 for the control group of children. The mean age of children in both the control group and the designated study subgroups did not differ statistically. The mean length of hospitalization was statistically significantly longer in the study group compared to the control group (5.1 ± 4.4 vs. 7.7 ± 2.8 days, respectively) (*p* < 0.001). Children with sepsis were hospitalized for 8.0 ± 2.8 days, while children with systemic inflammatory reaction and no diagnosis of SIRS for 6.2 ± 2.2 and 6.1 ± 2.7 days, respectively (*p* < 0.001).

Median serum concentrations of IL-6, sTREM-1, PCT, and CRP on the day of admission were statistically significantly higher in the study group compared to the control group (21.4 pg/ml, 18.7 pg/ml, 0.6 ng/ml, and 24.9 mg/l vs. 3.48 pg/ml, 18.7 pg/ml, 0.0 ng/ml, and 1.0 mg/l, respectively). There were statistically significant differences in median concentrations of IL-6 and PCT among the study group depending on the disease severity. The highest IL-6 median serum concentration was reported in the SIRS group (32.6 pg/ml (13.8-94.0 pg/ml)), followed by the sepsis and N-SIRS groups (26.4 pg/ml (11.5-71.4 pg/ml) and 18.1 pg/ml (8.5-29.2 pg/ml), respectively). In contrast to IL-6, median CRP and PCT serum concentrations were the lowest in the N-SIRS group and increased with disease severity, but only in the case of procalcitonin, the differences were statistically significant (*p* < 0.001). There were no statistically significant differences in sTREM-1 median serum concentrations between study subgroups (N-SIRS, SIRS, and sepsis subgroups) (Table 1).

Results of detailed analyses of differences in IL-6, sTREM-1, PCT, and CRP serum concentrations between subgroups are presented in Table 2. The median concentrations of IL-6, PCT, and CRP were statistically significantly higher in each study subgroup (N-SIRS, SIRS, and sepsis subgroups) compared to the control group. sTREM-1 median concentration was statistically significantly higher comparing the SIRS group with the control group, as well as the sepsis group with the control group. There were also statistically significant differences in IL-6, sTREM-1, and PCT median concentrations between the group of feverish patients without diagnosed SIRS (N-SIRS) and patients with SIRS/sepsis. There were no statistically significant differences between SIRS and sepsis groups in median concentrations of the analyzed markers.

Based on the ROC curves, the diagnostic value of IL-6, sTREM-1, PCT, and CRP in an acute inflammation diagnosis (especially in SIRS and sepsis) and the prognostic value of febrile disease course were evaluated.

**Figure 1:**
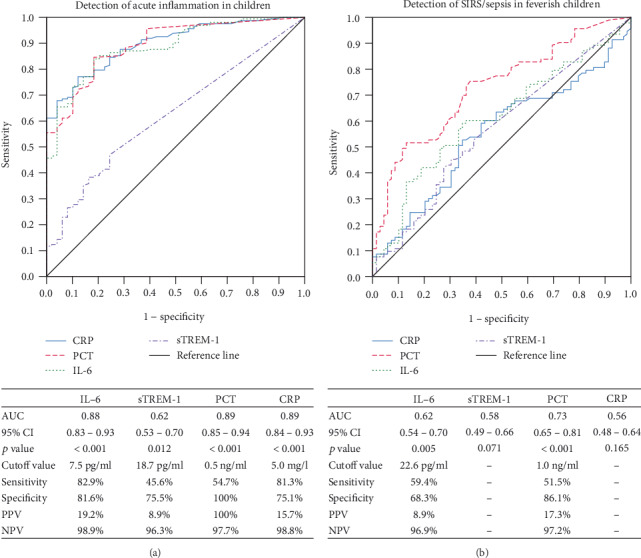
Diagnostic value of IL-6, sTREM-1, PCT, and CRP for diagnosis of acute inflammation (a) and SIRS/sepsis in feverish children (b).

The diagnostic usefulness of all the biomarkers was recognized for the diagnosis of acute inflammation in children. Among them, the highest sensitivity and specificity were found for IL-6. For the cutoff value of 7.5 pg/ml, the sensitivity was 82.9% and the specificity 81.6% (AUC 0.88). Of the analyzed biomarkers, procalcitonin and IL-6 had potential usefulness in the diagnosis of SRIS/sepsis in feverish children. For procalcitonin at the cutoff value of 1.0 ng/ml, the sensitivity was 51.5% and the specificity was 86.1% (AUC 0.73), while for IL-6 at the cutoff of value of 22.6 pg/ml, the sensitivity was 59.4% and the specificity was 68.3% (AUC 0.62) (Figure 1).

**Figure 2:**
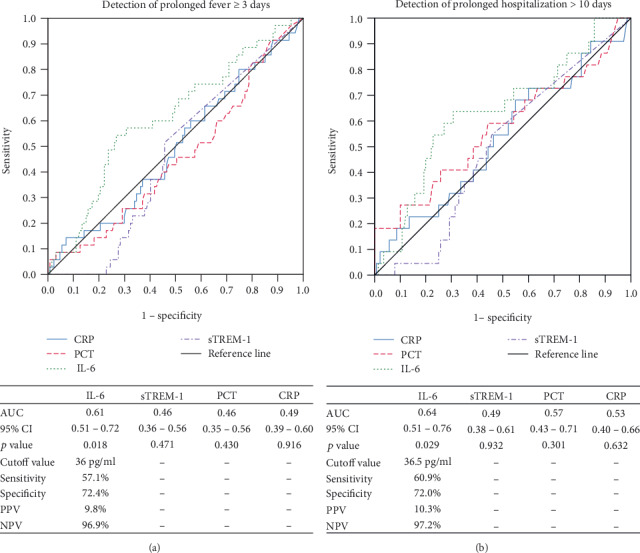
Prognostic value of IL-6, sTREM-1, PCT, and CRP of prolonged fever ≥ 3 days (a) and prolonged hospitalization > 10 days (b).

In order to assess the prognostic value of the analyzed markers, the relationship between the baseline concentration of IL-6, sTREM-1, PCT, and CRP and the duration of fever and length of hospitalization was analyzed. It was found that only IL-6 is a statistically significant prognostic marker of the febrile disease course, defined as prolonged fever ≥ 3 days and prolonged hospitalization > 10 days. For the prediction of prolonged fever ≥ 3 days at the cutoff value of 36 pg/ml, the sensitivity was 57.1% and the specificity was 72.4% (AUC 0. 61). In the case of prediction of hospitalization time > 10 days at the cutoff value of 36.5 pg/ml, the sensitivity was 60.9% and the specificity was 72.0% (AUC 0.64) (Figure 2).

## 4. Discussion

Our study confirmed the usefulness of IL-6, sTREM-1, PCT, and CRP in the diagnosis of acute inflammation in feverish children. The mean serum concentrations of all the analyzed biomarkers were statistically significantly higher in the study group compared to the control group. In further analyses, the diagnostic value of IL-6, sTREM-1, PCT, and CRP in the diagnosis of systemic inflammatory response syndrome and sepsis in feverish children was evaluated. Mean IL-6, sTREM-1, and PCT serum concentrations were statistically significantly higher in the group of patients with SIRS/sepsis compared to the group of feverish patients without diagnosed SIRS (N-SIRS). Based on the ROC curve analysis, it was shown that of all the biomarkers tested, only two—IL-6 and procalcitonin—had potential usefulness in the diagnosis of SIRS/sepsis in children with fever.

In the literature, most studies assessing the diagnostic value of IL-6 and sTREM-1 in SIRS/sepsis are related to adults [8, 23–27]. Available studies on children often concern selected groups of patients, such as newborns or children with febrile neutropenia, which makes it difficult to compare them with our results [20, 28–32].

In Lamping et al.'s study, 56 children with sepsis and 233 children with SIRS were hospitalized in the ICU. The median levels of IL-6, PCT, and CRP were statistically significantly higher in children with sepsis compared to children with SIRS (118 pg/ml, 2.55 ng/ml, and 48 mg/l vs. 52 pg/ml, 1.0 ng/ml, and 33.5 mg/l, respectively) [33]. In our own study, the median concentration of PCT and CRP was higher in the group of patients with sepsis compared to those with SIRS (1.15 ng/ml and 34.4 mg/l vs. 0.8 ng/ml and 28.7 mg/l, respectively), whereas IL-6 concentration was higher in the group of SIRS patients compared to children with sepsis (32.6 pg/ml vs. 26.4 pg/ml).

Promising results regarding the usefulness of sTREM-1 as a diagnostic and prognostic marker in sepsis were obtained by Adly et al. In a study including 112 newborns hospitalized due to early-onset sepsis (EOS) and late-onset sepsis (LOS), the initial mean sTREM-1 serum concentration was statistically significantly higher among newborns with sepsis compared to the control group. Adly et al. reported 100% sensitivity and specificity for sTREM-1 at the cutoff value of 310 pg/ml as a diagnostic marker for neonatal sepsis (AUC 1.0) [20]. In Sarafidis et al.'s study, which included 52 newborns, the usefulness of sTREM-1 compared to IL-6 in the LOS diagnostics was evaluated. Based on the ROC curve analysis, significant AUC values were found for both markers (0.733 and 0.892, respectively). Sensitivity and specificity of sTREM-1 as a diagnostic marker of neonatal sepsis at the cutoff value of 143 pg/ml were 70% and 71%, respectively. Interleukin-6 with a cutoff value of 66 pg/ml was characterized by 80% sensitivity and 81% specificity [28]. Ganesan et al. showed that in the diagnosis of sepsis in newborns, IL-6 is characterized by higher sensitivity, but also low specificity in comparison with CRP, respectively (100% and 62.9% vs. 80% and 65.7%). Coadministration of CRP and IL-6 was associated with an increase in specificity to 75.5% [29]. The high diagnostic value of procalcitonin in the course of EOS in newborns was confirmed by Nakstad. The sensitivity of PCT was 88% and the specificity was 93%, and it was higher in comparison with IL-6, respectively (80% and 88%). Similar to the results of Ganesan et al., the combined use of procalcitonin and interleukin-6 increased the diagnostic value of the analyzed markers [30].

According to some authors, sTREM-1, when used as a single marker, has an insufficient diagnostic value in the differentiation of sepsis from fever unrelated to bacteremia [34]. Pontrelli et al., based on a meta-analysis of 9 studies including 961 patients, showed that sTREM-1 is not a sufficiently sensitive and specific diagnostic marker of sepsis in children [35]. In our own study, sTREM-1 was also not useful in SIRS/sepsis diagnosis among feverish children, which may result from the study that was conducted among children hospitalized in the general pediatric ward. Data from the literature indicate that sTREM-1 concentrations are higher in patients with a more severe course of infection, hospitalized in intensive care units, and the results assessing the usefulness of sTREM-1 as a diagnostic marker of sepsis in these patients are more promising. Analyzing the results of our own study, it seems that IL-6 may be a marker of similar diagnostic value to procalcitonin. This is confirmed by the studies of other authors [30, 33, 36]. Undoubtedly, more research is needed to assess the diagnostic value of IL-6 and sTREM-1, including larger and more homogeneous groups of children hospitalized in different pediatric wards.

The results of studies assessing the prognostic value of interleukin-6 are not conclusive but indicate its potential usefulness as a prognostic marker in sepsis [37, 38]. Many studies also confirm the potential value of sTREM-1 as a prognostic marker of sepsis in adults [26, 37, 39–41].

The limited data from the literature regarding sepsis prognosis in children indicate a higher PCT value as compared to CRP and sTREM-1. According to Carrol et al.'s study, procalcitonin is the best predictor of death in children with serious bacterial infection (SBI), with 0.61 AUC. In the case of sTREM-1, the AUC was 0.54 and for CRP 0.43 [42].

Adly et al. observed a significant decrease in sTREM-1 serum concentration after 48 hours of antibiotic therapy, with significantly lower values in neonates with sterile control blood cultures, which may indicate the potential significance of sTREM-1 as a marker for monitoring the course of the disease. For sTREM-1 as a factor indicating a poor prognosis in the course of neonatal sepsis at the cutoff value of 1100 pg/ml, 100% sensitivity and 97% specificity (AUC 0.97) were reported [20].

In our own study, the prognostic value was assessed on the basis of the relationship between initial serum levels of the analyzed biomarkers and the duration of fever and length of hospitalization. Based on the ROC curve analysis, in both cases, it was found that only IL-6 is a statistically significant prognostic factor of prolonged fever exceeding 3 days and prolonged hospitalization over 10 days.

In conclusion, it seems that IL-6 may be a useful diagnostic and prognostic marker of SIRS/sepsis in children. The assessment of the usefulness of sTREM-1 in SIRS/sepsis diagnosis in feverish children requires further research, especially in the pediatric population.

## 5. Conclusions

1. Elevated levels of IL-6 and PCT are important risk factors for the development of SIRS/sepsis in children with fever
2. It seems that elevated IL-6 baseline serum level may predict a more severe course of febrile illness in children
3. The assessment of the usefulness of sTREM-1 in the diagnosis of SIRS/sepsis in feverish children requires further research

## Figures and Tables

**Table 1:** Demographic and clinical characteristics in the study and control groups.

Characteristics	Study group (*n* = 180)	Control group (*n* = 49)	*p* value	Study group			*p* value
				N-SIRS (*n* = 82)	SIRS (*n* = 45)	Sepsis (*n* = 53)	
Sex							
Male	101 (56.1)	28 (57.1)	0.897	45 (54.9)	34 (64.2)	22 (48.9)	0.302
Female	79 (43.9)	21 (42.9)		37 (45.1)	19 (35.8)	23 (51.1)	
Age (years)	6.4 ± 3.2	7.1 ± 5.2	0.549	6.2 ± 4.9	7.3 ± 3.0	6.7 ± 3.8	0.587
Hospitalization (days)	7.7 ± 2.8	5.1 ± 4.4	<0.001	6.1 ± 2.7	6.2 ± 2.2	8 ± 2.8	<0.001
Prolonged fever ≥ 3 days after admission	1.6 ± 1.4	—	—	1.4 ± 1.3	1.5 ± 1.1	1.9 ± 1.3	0.154
CRP (mg/l)	24.9 (7.6-65.4)	1 (0.3-4.2)	<0.001	17.9 (7.9-58.9)	28.7 (6.9-71.8)	34.4 (8.7-74.2)	0.243
PCT (ng/ml)	0.6 (0.2-1.6)	0 (0-0.1)	<0.001	0.31 (0.1-0.2)	0.8 (0.341.7)	1.15 (0.5-3.0)	<0.001
IL-6 (pg/ml)	21.4 (10.5-53.3)	3.48 (1.8-6.2)	<0.001	18.1 (8.5-29.2)	32.6 (13.8-94.0)	26.4 (11.5-71.4)	0.013
sTREM-1 (pg/ml)	18.7 (18.7-34.3)	18.7 (18.7-18.7)	0.005	18.7 (18.7-27.4)	18.7 (18.7-27.3)	21.8 (18.7-40.2)	0.098

Quantitative data are expressed as mean ± SD or median (interquartile range). Qualitative data are expressed as number (percentage). CRP: C-reactive protein; PCT: procalcitonin; IL-6: interleukin-6; sTREM-1: soluble triggering receptor expressed on myeloid cells-1.

**Table 2:** Differences in serum concentrations of CRP, PCT, IL-6, and sTREM-1 between the analyzed subgroups.

	Control vs. N-SIRS	Control vs. SIRS	Control vs. sepsis	N-SIRS vs. SIRS/sepsis	SIRS vs. sepsis
	*p* value				
CRP (mg/l)	<0.001	<0.001	<0.001	0.165	0.414
PCT (ng/ml)	0.008	<0.001	<0.001	<0.001	0.12
IL-6 (pg/ml)	<0.001	<0.001	<0.001	0.005	0.39
sTREM-1 (pg/ml)	0.101	0.013	0.001	0.048	0.389

## Data Availability

All data are stored with the corresponding author.

## References

[1] Bone R. C., Balk R. A., Cerra F. B. (1992). American College of Chest Physicians/Society of Critical Care Medicine consensus conference: definitions for sepsis and organ failure and guidelines for the use of innovative therapies in sepsis. *Critical Care Medicine*.

[2] Vincent J. L., Opal S. M., Marshall J. C., Tracey K. J. (2013). Sepsis definitions: time for change. *The Lancet*.

[3] Singer M., Deutschman C. S., Seymour C. W. (2016). The Third International Consensus Definitions for Sepsis and Septic Shock (Sepsis-3). *JAMA*.

[4] Simon L., Saint-Louis P., Amre D. K., Lacroix J., Gauvin F. (2008). Procalcitonin and C-reactive protein as markers of bacterial infection in critically ill children at onset of systemic inflammatory response syndrome. *Pediatric Critical Care Medicine*.

[5] Toikka P., Irjala K., Juven T. (2000). Serum procalcitonin, C-reactive protein and interleukin-6 for distinguishing bacterial and viral pneumonia in children. *The Pediatric Infectious Disease Journal*.

[6] Gendrel D., Raymond J., Coste J. (1999). Comparison of procalcitonin with C-reactive protein, interleukin 6 and interferon-alpha for differentiation of bacterial vs. viral infections. *The Pediatric Infectious Disease Journal*.

[7] Tang B. M., Eslick G. D., Craig J. C., McLean A. S. (2007). Accuracy of procalcitonin for sepsis diagnosis in critically ill patients: systematic review and meta-analysis. *Lancet Infectious Diseases*.

[8] Castelli G. P., Pognani C., Cita M., Stuani A., Sgarbi L., Paladini R. (2006). Procalcitonin, C-reactive protein, white blood cells and SOFA score in ICU: diagnosis and monitoring of sepsis. *Minerva Anestesiologica*.

[9] Barton B. E. (1996). The biological effects of interleukin 6. *Medicinal Research Reviews*.

[10] Gao J., Zhang A. Q., Pan W. (2015). Association between IL-6-174G/C polymorphism and the risk of sepsis and mortality: a systematic review and meta-analysis. *PLoS One*.

[11] Sikora J. P., Chlebna-Sokol D., Krzyzanska-Oberbek A. (2001). Proinflammatory cytokines (IL-6, IL-8), cytokine inhibitors (IL-6sR, sTNFRII) and anti-inflammatory cytokines (IL-10, IL-13) in the pathogenesis of sepsis in newborns and infants. *Archivum Immunologiae et Therapiae Experimentalis*.

[12] Hunter C. A., Jones S. A. (2015). IL-6 as a keystone cytokine in health and disease. *Nature Immunology*.

[13] Alqahtani M. F., Marsillio L. E., Rozenfeld R. A. (2014). A review of biomarkers and physiomarkers in pediatric sepsis. *Clinical Pediatric Emergency Medicine*.

[14] Bouchon A., Dietrich J., Colonna M. (2000). Cutting edge: inflammatory responses can be triggered by TREM-1, a novel receptor expressed on neutrophils and monocytes. *Journal of Immunology*.

[15] Colonna M., Facchetti F. (2003). TREM-1 (triggering receptor expressed on myeloid cells): a new player in acute inflammatory responses. *The Journal of Infectious Diseases*.

[16] Tessarz A. S., Cerwenka A. (2008). The TREM-1/DAP12 pathway. *Immunology Letters*.

[17] Henriquez-Camacho C., Losa J. (2014). Biomarkers for Sepsis. *BioMed Research International*.

[18] Gibot S. (2005). Clinical review: role of triggering receptor expressed on myeloid cells-1 during sepsis. *Critical Care*.

[19] Gibot S., Cravoisy A. (2004). Soluble form of the triggering receptor expressed on myeloid cells-1 as a marker of microbial infection. *Clinical Medicine & Research*.

[20] Adly A. A. M., Ismail E. A., Andrawes N. G., El-Saadany M. A. (2014). Circulating soluble triggering receptor expressed on myeloid cells-1 (sTREM-1) as diagnostic and prognostic marker in neonatal sepsis. *Cytokine*.

[21] Tammaro A., Derive M., Gibot S., Leemans J. C., Florquin S., Dessing M. C. (2017). TREM-1 and its potential ligands in non-infectious diseases: from biology to clinical perspectives. *Pharmacology & Therapeutics*.

[22] Goldstein B., Giroir B., Randolph A., the Members of the International Consensus Conference on Pediatric Sepsis (2005). International pediatric sepsis consensus conference: definitions for sepsis and organ dysfunction in pediatrics. *Pediatric Critical Care Medicine*.

[23] Wu Y., Wang F., Fan X. (2012). Accuracy of plasma sTREM-1 for sepsis diagnosis in systemic inflammatory patients: a systematic review and meta-analysis. *Critical Care*.

[24] Gibot S., Kolopp-Sarda M.-N., Béné M. C. (2004). Plasma level of a triggering receptor expressed on myeloid cells-1: its diagnostic accuracy in patients with suspected sepsis. *Annals of Internal Medicine*.

[25] Su L., Han B., Liu C. (2012). Value of soluble TREM-1, procalcitonin, and C-reactive protein serum levels as biomarkers for detecting bacteremia among sepsis patients with new fever in intensive care units: a prospective cohort study. *BMC Infectious Diseases*.

[26] Wang H., Chen B. (2011). Diagnostic role of soluble triggering receptor expressed on myeloid cell-1 in patients with sepsis. *World Journal of Emergency Medicine*.

[27] Bayram H., Tünger Ö., Çivi M. (2015). Diagnostic and prognostic value of procalcitonin and sTREM-1 levels in sepsis. *Turkish Journal of Medical Sciences*.

[28] Sarafidis K., Soubasi-Griva V., Piretzi K. (2010). Diagnostic utility of elevated serum soluble triggering receptor expressed on myeloid cells (sTREM)-1 in infected neonates. *Intensive Care Medicine*.

[29] Ganesan P., Shanmugam P., Sattar S. B. A., Shankar S. L. (2016). Evaluation of IL-6, CRP and hs-CRP as early markers of neonatal sepsis. *Journal of Clinical and Diagnosis Research*.

[30] Nakstad B. (2018). The diagnostic utility of procalcitonin, interleukin-6 and interleukin-8, and hyaluronic acid in the Norwegian consensus definition for early-onset neonatal sepsis (EONS). *Infection and Drug Resistance*.

[31] Arzanian M. T., Soltani B., Fahimzad A., Shiva F., Shamshiri A. R., Karimi A. (2011). Association of serum soluble triggering receptor expressed on myeloid cells levels in malignant febrile neutropenic patients with bacteremia and fungemia. *Iranian Journal of Pediatrics*.

[32] Miedema K. G. E., de Bont E. S. J. M., Elferink R. F. M. O. (2011). The diagnostic value of CRP, IL-8, PCT and sTREM-1 in the detection of bacterial infections in pediatric oncology patients with febrile neutropenia. *Support Care Cancer*.

[33] Lamping F., Jack T., Rübsamen N. (2018). Development and validation of a diagnostic model for early differentiation of sepsis and non-infectious SIRS in critically ill children – a data-driven approach using machine-learning algorithms. *BMC Pediatrics*.

[34] Kevan E. N., Simmons J. R., Kocoshis S. A., Cohen M. B., Rudolph J. A. (2011). sTREM-1 and LBP in central venous catheter-associated bloodstream infections in pediatric intestinal failure. *Journal of Pediatric Gastroenterology and Nutrition*.

[35] Pontrelli G., de Crescenzo F., Buzzetti R. (2016). Diagnostic value of soluble triggering receptor expressed on myeloid cells in paediatric sepsis: a systematic review. *Italian Journal of Pediatrics*.

[36] Hou T., Huang D., Zeng R., Ye Z., Zhang Y. (2015). Accuracy of serum interleukin (IL)-6 in sepsis diagnosis: a systematic review and meta-analysis. *International Journal of Clinical and Experimental Medicine*.

[37] Li Z., Wang H., Liu J., Chen B., Li G. (2014). Serum soluble triggering receptor expressed on myeloid cells-1 and procalcitonin can reflect sepsis severity and predict prognosis: a prospective cohort study. *Mediators of Inflammation*.

[38] Oda S., Hirasawa H., Shiga H., Nakanishi K., Matsuda K., Nakamua M. (2005). Sequential measurement of IL-6 blood levels in patients with systemic inflammatory response syndrome (SIRS)/sepsis. *Cytokine*.

[39] Zhang J., She D., Feng D., Jia Y., Xie L. (2011). Dynamic changes of serum soluble triggering receptor expressed on myeloid cells-1 (sTREM-1) reflect sepsis severity and can predict prognosis: a prospective study. *BMC Infectious Diseases*.

[40] Su L., Liu D., Chai W., Liu D., Long Y. (2016). Role of sTREM-1 in predicting mortality of infection: a systematic review and meta-analysis. *BMJ Open*.

[41] Oku R., Oda S., Nakada T. (2013). Differential pattern of cell-surface and soluble TREM-1 between sepsis and SIRS. *Cytokine*.

[42] Carrol E. D., Mankhambo L. A., Jeffers G. (2009). The diagnostic and prognostic accuracy of five markers of serious bacterial infection in Malawian children with signs of severe infection. *PLoS One*.

